# SCOP2 prototype: a new approach to protein structure mining

**DOI:** 10.1093/nar/gkt1242

**Published:** 2013-11-29

**Authors:** Antonina Andreeva, Dave Howorth, Cyrus Chothia, Eugene Kulesha, Alexey G. Murzin

**Affiliations:** ^1^MRC Laboratory of Molecular Biology, Francis Crick Avenue, Cambridge, CB2 0QH, UK and ^2^European Bioinformatics Institute, Hinxton, Cambridge, CB10 1SD, UK

## Abstract

We present a prototype of a new structural classification of proteins, SCOP2 (http://scop2.mrc-lmb.cam.ac.uk/), that we have developed recently. SCOP2 is a successor to the Structural Classification of Proteins (SCOP, http://scop.mrc-lmb.cam.ac.uk/scop/) database. Similarly to SCOP, the main focus of SCOP2 is to organize structurally characterized proteins according to their structural and evolutionary relationships. SCOP2 was designed to provide a more advanced framework for protein structure annotation and classification. It defines a new approach to the classification of proteins that is essentially different from SCOP, but retains its best features. The SCOP2 classification is described in terms of a directed acyclic graph in which nodes form a complex network of many-to-many relationships and are represented by a region of protein structure and sequence. The new classification project is expected to ensure new advances in the field and open new areas of research.

## INTRODUCTION

Nearly two decades have passed since the Structural Classification of Proteins (SCOP) database was created at the MRC Laboratory of Molecular Biology and Centre of Protein Engineering in Cambridge (1). The SCOP project has brought together a number of previous studies on the principles of protein structure and evolution (2–9) (*more references are available at* http://scop.mrc-lmb.cam.ac.uk/scop/refs.html). Over time SCOP and similar structural classifications such as CATH (10) have become valuable resources for many areas of protein research.

The notion of protein evolution, embodied in SCOP, allowed the discrete grouping of proteins based not only on their structural similarity but also on their probable evolutionary origin. Like in the Linnaean taxonomy, discrete units, *domains*, were grouped hierarchically on the basis of their common structural and evolutionary relationships. In accordance with the degree of evolutionary divergence and, respectively, structural (dis)similarity, SCOP organized protein domains into *families* and *superfamilies*. These were further grouped into structural *folds*, which were not necessarily indicative of a common evolutionary origin and *classes* that reflect the domains' secondary structures. Each grouping in the classification was the product of a careful case-by-case analysis of protein structures and a detailed knowledge of protein function and evolution (11–14).

The original tree-like SCOP classification was based on several assumptions: (i) sequences of proteins performing the same molecular function have diverged with speciation of the organisms; (ii) a given protein sequence can have only one folded ‘native’ structure; (iii) homologous proteins fold into similar structures; (iv) protein structures are evolutionarily more conserved than sequences; and (v) proteins of independent evolutionary lineages can share a common fold. In summary, it was thought that protein fold is physically and evolutionarily invariant.

The primary purpose of SCOP was to assist structural biologists in the analysis and exploration of proteins’ structural similarities. The simple hierarchical classification supported the development of tools and algorithms and it was successfully used by many applications. It also contributed to our understanding of protein repertoire, of how proteins relate to each other and how their structures and functions evolved. The database was applied to other areas of protein research such as protein structure prediction and large-scale genome analyses and annotations (15). SCOP has also been used for matching sequence-based to structure-based domains (16), prediction of protein–protein interactions (17), matching protein structure with enzymatic activity (18) and other studies. SCOP also prompted the development of automated classifications such as SCOPmap (19) and QSCOP (20). These additional uses of SCOP for which the database was not originally designed caused numerous database revisions and the development of additional features imposing stricter criteria and stable definitions (21,22). As a result, updating SCOP became more time-consuming and the level of inconsistency increased with the amount of data while attempting to satisfy different users’ demands at once.

The simple taxonomy-like classification of protein structures was useful when the amount of data was moderate. However, with the increased amount of structural data it has become clear that relationships between proteins are more complex than anticipated and that protein evolutionary pathways do not always conform to the same rules (23–26). The vast amount of structural information also provided new insights into the mechanisms underlying molecular recognition and evolution of protein structure. In turn, many theories now need to be revisited since exceptions to the classic (empirical) rules have been observed (27–29). The simple SCOP classification scheme was unable to represent new discoveries and findings or to recreate some of the complex scenarios of protein evolution. It also has become clear that the classification scheme cannot be mended by further modifications and adjustments but it requires fundamental redesign. Therefore, we endeavor to develop a more advanced framework for presentation of protein relationships, a new classification scheme that can be adapted to any particular case and evolutionary scenario.

We have constructed a prototype of the new SCOP, named SCOP2. In essence, the SCOP2 prototype defines a new approach to the classification of proteins and aims to tackle the obstacles and inconsistencies that arose from the numerous examples of non-trivial protein relationships. SCOP2 retains the best features of the old SCOP database but it differs in several key aspects and provides new data not available in the old resource. Taken together, the new classification project is expected to ensure new advances in the field and open new directions of research.

## OVERVIEW OF SCOP2

**Figure 1.**
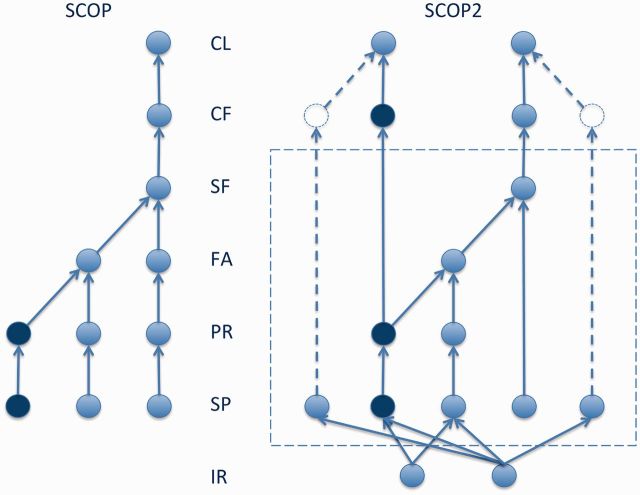
SCOP and SCOP2 graphs compared. (Left) A section of the SCOP hierarchical tree, showing the classification into the six obligatory levels: *Protein species* (SP), *Protein* (PR), *Family* (FA), *Superfamily* (SF), *Fold* (CF) and *Class* (CL). The homologous proteins with distinct folds, e.g., the Cro repressors (*25*), are compulsorily assigned in the same family and so progressively to the same superfamily, fold and class. There is an obligatory node at every level from the root to a leaf, even if such node does not represent any actual relationship, e.g., ‘singleton’ protein family consisting of a single protein that only has a relationship with itself. (Right) In SCOP2, the structural and evolutionary relationships are separated, allowing the classification of the homologous proteins into different folds and structural classes while keeping them in the same evolutionary family and superfamily. Non-obligatory single-child nodes are skipped to emphasize that relatives of a ‘singleton’ protein exist only at the superfamily level. The new category specific to SCOP2, *'other' relationships* (IR), is also shown on the graph. These relationships include but are not limited to non-hierarchical relationships between homologous and non-homologous proteins with different folds sharing a large common substructure or motif (see also Figure 2).

Similarly to SCOP, the main focus of SCOP2 is on knowledge-based expert analysis and classification of proteins that are structurally characterized and deposited in the Protein Data Bank, PDB (30). Proteins are organized according to their structural and evolutionary relationships, but, in contrast to SCOP, instead of a simple tree-like hierarchy these relationships form a complex network of nodes (Figure 1). The classification of proteins is described in terms of a directed acyclic graph in which each node defines a relationship of particular type and is exemplified by a region of protein structure and sequence. Importantly, there can be more than one parental node for a child node that allows multiple routes to a particular relationship.

The relationships in SCOP2 fall into four major categories, two of which, *Protein types* and *Evolutionary events*, do not have counterparts in SCOP. In the *Protein types* category, proteins are divided into four main types: soluble, membrane, fibrous and intrinsically disordered, each of which to a large extent correlates with characteristic sequence and structural features. Classification of proteins according to their types resolves the inconsistent classification of membrane and coiled coil proteins, previously organized in their own classes, and allows placing them in the correct structural classes. Proteins belonging to different types, e.g., soluble and membrane proteins, can now share a common fold and even can be homologous to each other. The other category, *Evolutionary events* is introduced to facilitate the annotation of various structural rearrangements and peculiarities that have been observed among related proteins and which have given rise to substantial structural differences.

Third of the four major categories in SCOP2, the *Structural classes*, organizes protein folds strictly according to their secondary structural content. They now strictly correspond to the structural classes previously defined by Levitt and Chothia (2). In SCOP2 the structural class becomes an attribute of protein fold and therefore is independent of the protein evolutionary relationships. Similar protein folds but with different secondary structural content are placed into different classes.

The fourth main category in SCOP2, the protein relationships, consists of three subcategories: *Structural*, *Evolutionary* and ‘*Other*’ relationships. The ‘other’ relationships, a category unique to SCOP2, aims to define and annotate relationships such as internal structural repeats, common motifs and subfolds that have not been a subject of classification in the SCOP database.

SCOP2 retains the evolutionary levels of SCOP, *Species*, *Protein*, *Family*, and *Superfamily* but their content and definitions are different. *Species* corresponds to the individual gene product and is represented by its full-length sequence, *Protein* groups together orthologous proteins and is defined as a subsequence that can be found on its own, *Family* corresponds to the conserved sequence region shared by closely related proteins and *Superfamily* is represented by the common structural region shared by different protein families. Importantly, the domains representing *Family* and *Superfamily* levels can span over more than one structural domain. In addition to these levels, SCOP2 contains a new level, *Hyperfamily.* The *Hyperfamily* level is introduced mainly to deal with the most populated and structurally diverse SCOP superfamilies. One of the striking differences between SCOP and SCOP2 is that these distinct levels are not obligatory; e.g., family could be the highest evolutionary relationship to which a protein domain belongs since there are no other distantly related protein domains that could form a superfamily.

Due to the constraints of the old SCOP scheme in which structural relationships reside at the top of the hierarchy, some homologous proteins were classified into the same fold even if they have different folds (Figure 1). In SCOP2, the protein structural and evolutionary relationships are presented in separate branches (categories) to ensure more consistent classification of evolutionarily related but structurally distinct proteins. Thus, these members of a superfamily that have undergone evolutionary events affecting their protein structure will be classified into different folds. Protein fold in SCOP2 is an attribute of structural domain and is defined strictly on the basis of global structural features as originally described (31).

In SCOP2, there is no *a priori* division of protein structures into domains, in which one domain size fits all possible relationships. The protein domain is defined as a unit of relationship and its boundaries are dependent on a given relationship. The SCOP2 classification is based on representative sequences and structures. The manual annotation of these representatives is then automatically extended to related entries. Additional annotation of the SCOP2 entries is provided using a controlled vocabulary (keywords and tags). This allows easy retrieval of a subset of proteins with a given feature or automatic processing of the classification data.

## WEB-BASED INTERFACE

**Figure 2.**
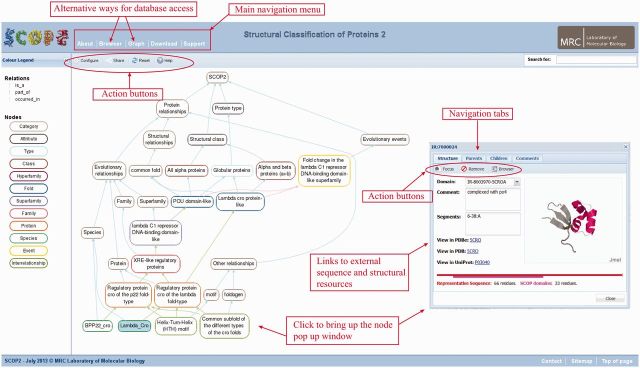
Exploring SCOP2 with a custom graph viewer tool. A screenshot of a SCOP2-graph webpage showing a combined ancestor chart of two orthologous Cro repressors from bacteriophages lambda and p22. (The URL to view this page is http://scop2.mrc-lmb.cam.ac.uk/graph/example1). In addition to the protein relationships, schematically shown in Figure 1, the chart includes additional ‘ontology’ categories. The displayed chart can be expanded or collapsed via the node popup window. The node popup window also displays additional information about the selected node, in particular the associated structural and sequence domains and their boundaries representing the relationship. In this example, the domain, representing the common subfold of the different Cro types, is shown in the context of the full-length protein sequence and structure.

The database is available over the web from http://scop2.mrc-lmb.cam.ac.uk/. The SCOP2 data can be accessed in two different ways: via SCOP2-browser and SCOP2-graph. SCOP2-browser allows navigation through the SCOP2 classification in a traditional way by browsing pages displaying the node information. An alternative way of viewing the SCOP2 data is by using the SCOP2-graph (Figure 2). SCOP2-graph is a web-based viewer for display and navigation through the graph of SCOP2 nodes. The graph display is based on GraphViz software (32). Each navigation tool, SCOP2-browser and SCOP2-graph, has a search engine allowing the retrieval of any data of interest. Each tool also provides hyperlinks to external databases such as PDB and Uniprot and to the original SCOP entries. The 3D structures of protein regions representing different SCOP2 relationships are visualized with Jmol and Rasmol. All SCOP2 data are stored in a relational MySQL database, which is available for download in addition to parseable files.

## FUTURE DEVELOPMENTS

During the development of the SCOP2 prototype, we tried to make use of the knowledge that we have acquired over the past years and the lessons we have learned during the classification of protein structures. We believe that there are many peculiarities of proteins and their structures that have been missed due to the constraints of the original SCOP hierarchical schema. We also hope that the new resource will prove to be useful and that it could open new areas for protein analysis and research.

We are aware that many applications have been built that are using the SCOP database. Therefore, we begin introducing the new database with the release of its working prototype. It contains only a small fraction of the available structural data that, however, can provide a clear view of the SCOP2 relationships and their complexity. The SCOP2 prototype provides links to the original SCOP entries from which the SCOP2 entries were derived and hence creates a possibility for the users to compare both databases. We ask for comprehensive feedback from our users that would guide us for future database development and expansion. We attempt to gradually convert old SCOP data while tracking their history where possible. We will also update the database with new data, giving priority to representatives of new families in the PDB.

## FUNDING

Medical Research Council (MRC) [MC_U105192716]; Biotechnology and Biological Sciences Research Council (in part) [BB/I024917/1]. Funding for open access charge: The MRC core funding.

*Conflict of interest statement*. None declared.
