# SCOP2 prototype: a new approach to protein structure mining

**DOI:** 10.1093/nar/gku841

**Published:** 2014-10-07

SCOP2 prototype: a new approach to protein structure mining.

Antonina Andreeva, Dave Howorth, Cyrus Chothia, Eugene Kulesha and Alexey G. Muzin

Nucl. Acids Res. (2014) 42 (D1): D310-D314. doi: 10.1093/nar/gkt1242.

In the above article one relevant funding source mistakenly has been omitted. The correct funding statement is below.

Medical Research Council (MRC) [MC_U105192716]; Biotechnology and Biological Sciences Research Council (in part) [BB/I024917/1]; National Institutes of Health [R01-GM073109] through the US Department of Energy under Contract No. DE-AC02-05CH11231 (subcontract to A.G.M.; no financial support was drawn). Funding for open access charge: The MRC core funding.

